# Honey as a Sugar Substitute in Gluten-Free Bread Production

**DOI:** 10.3390/foods13182973

**Published:** 2024-09-19

**Authors:** Michela Cannas, Costantino Fadda, Pietro Paolo Urgeghe, Antonio Piga, Paola Conte

**Affiliations:** Department of Agricultural Sciences, Università degli Studi di Sassari, Viale Italia 39/A, 07100 Sassari, Italy; mcannas@uniss.it (M.C.); cfadda@uniss.it (C.F.); paolou@uniss.it (P.P.U.); pconte@uniss.it (P.C.)

**Keywords:** gluten-free bread, honey, physical properties, rheological properties, sucrose

## Abstract

In recent years, there has been a significant focus on enhancing the overall quality of gluten-free breads by incorporating natural and healthy compounds to meet consumer expectations regarding texture, flavor, and nutritional value. Considering the high glycemic index associated with gluten-free products, the use of honey, renowned for its numerous health benefits, may serve as an optimal alternative to sucrose. This study investigates the impact of substituting sucrose, either partially (50%) or entirely (100%), with five Sardinian honeys (commercial multifloral honey, cardoon, eucalyptus, and strawberry tree unifloral honeys, and eucalyptus honeydew honey), on the rheological properties of the doughs and the physico-chemical and technological properties of the resulting gluten-free breads. The results demonstrated that an optimal balance was achieved between the leavening and viscoelastic properties of the doughs and the physical and textural attributes of the resulting breads in gluten-free samples prepared with a partial substitution of cardoon and multifloral honeys. Conversely, the least favorable outcomes were observed in samples prepared with strawberry tree honey and eucalyptus honeydew honey at both substitution levels. Therefore, the different behavior observed among all honey-enriched gluten-free breads was likely attributable to the distinct botanical origins of honey rather than to the substitution percentages employed.

## 1. Introduction

Gluten represents a protein ingredient of paramount importance for the production of different items due to its viscoelastic properties, which are particularly relevant for bakery foods and specifically in bread, where it is responsible for the final porous structure [1]. The specific composition of gluten, which is characterized by a high content of prolamines and glutenins, facilitates the formation of a three-dimensional network, resulting in dough with unique properties of elasticity and resistance [2]. However, this same composition can also elicit allergic reactions or autoimmune disorders, collectively known as gluten-related disorders (GRDs). The most prevalent of these is celiac disease [3]. Intolerance to gluten can be avoided only with a long-life gluten-free (GF) diet. Consequently, it is necessary to eliminate from the diet all foods containing wheat, rye, barley, and oats, as well as any products that may contain gluten. The market for GF products is constantly upsurging, driven by an increase in the number of individuals diagnosed with GRDs and the number of consumers adopting a GF diet [4]. 

However, despite the extensive academic and industrial research for the development of GF products, especially bread, the quality of the commercially available products is lower than that of their gluten-containing counterparts. Indeed, GF bread is characterized by its lack of volume and texture, as well as a diminished nutritional value and an accelerated rate of staling [5]. The basic flours used in the production of GF breads include rice, corn, sorghum, and other GF cereals and starches, as well as pseudocereal flours, such as amaranth [6,7] and buckwheat [8], and legumes, such as chickpea [9] and soybean [10]. Moreover, in order to obtain a sufficient volume, crumb softness, and delay staling, additional ingredients with technological and nutritional functionality are required. These include hydrocolloids [11,12,13], fibers [14,15], chia and flax seeds [16,17], *psyllium* [18], proteins of different origin [19], enzymes [20], and modified starches [21]. Other recent approaches consider the use of sourdough obtained from GF flour, which may result in sensory improvement and shelf life extension of GF bread [22]. Emulsifiers are also important ingredients in GF dough development due to their softening effect on the crumb [23]. 

Of the ingredients described above, a typical GF formulation comprises the use of salt, yeast, and sugar. The latter plays a fundamental role, influencing the structure, color, shelf life, and sensory properties of the finished product. Fermentable sugars, including glucose and fructose (but also maltose and sucrose after enzymatic hydrolysis), are used by yeasts during the leavening process for their growth, allowing the production of carbon dioxide and, consequently, an increase in the volume of the dough and the finished product [24].

Furthermore, their ability to modulate the viscosity of the dough enables a more stable and homogeneous distribution of the gases produced during fermentation, thereby facilitating the formation of an alveolar structure in the crumb that is more aerated and uniform. Moreover, residual reducing sugars during cooking favor the non-enzymatic browning reactions, such as caramelization and the Maillard reaction (between sugars and amino acids), which positively influence the crust color and the aroma of the finished product. Finally, during storage, the high hygroscopicity of some types of sugars (especially levulose, invert sugar, and honey sugars) can extend the shelf-life of the bread. Sucrose is the main sugar used in the formulation of GF bread doughs at a rate of 5% or more on a flour basis [25]. Despite the beneficial technological and sensory effects previously reported, the use of sucrose further increases the already high glycemic index of GF bread. Therefore, finding a suitable substitute for sucrose is desirable. 

In this context, honey could be an excellent alternative to sucrose in the preparation of GF bread, thanks to its greater sweetening power, greater concentration of fructose, and lower glycemic index. In fact, for millennia, honey has been the only concentrated sugary food for which human health benefits have been recognized, as its use has been associated with immunostimulant and anti-inflammatory effects [26]. In particular, the incorporation of honey into the formulation of baked products, such as bread, enables the performance of a series of technological functions at both the level of the dough and of the finished product. In fact, the addition of honey to the formulation appears to enhance the dough’s ability to bind water (water-binding capacity), its tolerance during kneading, and the reduction in development and stability time. At the level of the finished product, it appears to enhance the specific volume and softness of the crumb, the darkening of the color of the crust, and the delayed staling of the final product [27].

In light of these considerations, the present study attempted to develop a qualitatively improved GF bread by including a natural product such as honey in the basic formulation. Based on what has been reported in the literature, the replacement of one type of sugar with another in the development of a food product aims to identify an adequate level of substitution that allows for the maintenance of the product’s technological quality while simultaneously improving its nutritional and sensorial profile [27,28]. 

To this end, three types of unifloral honeys (cardoon, eucalyptus, and strawberry tree), honeydew honey (eucalyptus), and a commercial multifloral honey were used as natural ingredients to replace sucrose in a conventional GF bread formulation. Specifically, the objective of this research was to evaluate the effects of partial (50%) or total (100%) replacement of sucrose with the five above-cited types of honey on the rheological characteristics of the intermediate products and on the technological quality of the final product. 

## 2. Materials and Methods

### 2.1. Raw Materials

Commercial rice flour, corn starch, guar gum, and Psyllium fiber were obtained from Chimab Campodarsego (Padova, Italy). Multifloral (MF), cardoon (CA), strawberry tree (ST), eucalyptus (EU), and honeydew eucalyptus (HE) honeys were produced in Sardinia in the Jerzu (Nu) area and kindly provided by the Plant Pathology and Entomology section of the Department of Agriculture of the University of Sassari. Honeys were produced in 2022. Fresh compressed yeast, salt, and sugar were purchased from a local supermarket. Sunflower oil was from Carapelli Firenze (Florence, Italy).

### 2.2. Breadmaking Process

All ingredients used to make both control and supplemented breads were based on 100 g of flour/starch (50% rice flour and 50% corn starch) and 90 mL of water (26 °C). The other ingredients were: 5% sunflower oil, 3% yeast, 5% sucrose, 1.8% sodium chloride, 1.5% guar gum, and 1.5% Psyllium fiber. A French bread type was produced.

Honey was incorporated into the basic formula at 2 different levels of supplementation, 2.5 and 5% (corresponding to 50% and 100% of sucrose substitution), as follows: control (CTRL), MF2.5%, MF5.0%, CA2.5%, CA5.0%, ST2.5%, ST5.0%, EU2.5%, EU5.0%, HE2.5%, and HE5.0%. GF breads were prepared using a straight-dough breadmaking process according to the procedure previously described by [29]. A total of four loaves of bread were produced for each formulation and production batch (*n* = 2).

### 2.3. Determination of Moisture Content of Honeys

The measurement of the water content of the honeys was carried out following the official method reported in the [30].

In general, the determination of the water content of honey is carried out indirectly by means of the refractometric method. This method is based on the principle that the refractive index of honey and, therefore, its solute content, varies as a function of humidity. At the same temperature, a higher humidity corresponds to a lower solute concentration. Thus, using the Wedmore formula, which relates the refractive index measured at 20 °C to the water content, it is possible to obtain the humidity value of honey expressed as a percentage.

For this purpose, an aliquot taken from each honey sample was placed inside a closed, perfectly sealed glass tube and heated in a thermostatic bath at a temperature of 50 °C ± 2 (WB- MF24, Falc Instruments, Treviglio, Italy) until the complete dissolution of any crystals present in the honey samples. Before proceeding with the measurement, performed using a digital refractometer (“Palette” PR-301, ATAGO CO, Ltd., Tokyo, Japan) covering high sugar concentrations (from 45 to 90 °Brix), the samples were allowed to cool to room temperature and carefully homogenized. The values obtained, expressed in °Brix (or mass percentage of sucrose), were converted into the corresponding refractive index values in accordance with the relationship established by the International Committee of Uniform Method of Sugar Analysis (ICUMSA) in the 16th session of 1974. Once the refractive index was obtained, the water content of the different honeys under study was calculated using Wedmore’s formula. It is important to highlight that, according to the Italian current legislation (law decree n. 179 of 21 May 2004), honey must have a water content of less than 20% in order to be marketed. The analysis was performed in triplicate for each honey sample.

### 2.4. Determination of Glucose and Fructose Content in Honey Samples

The glucose and fructose content of honey samples was determined using an Agilent 1100 series HPLC (Agilent Technologies, Santa Clara, CA, USA) equipped with an autosampler (G1313A), thermostated column compartment (G1316A) operating at 80 °C, quaternary pump (G1311A), and refractive index detector (G1362A). The chromatographic separation was performed using a Rezex RCM Ca^2+^ column (300 × 7.8 m) as the stationary phase and deionized water as the mobile phase. The flow rate was set to 0.6 mL/min, and the injection volume was 20 μL. Under the specified conditions, glucose and fructose had average retention times of 11.63 and 14.55, respectively. Quantification was performed by external calibration, and the results (means of three replicates) were expressed as g 100 g^−1^ of honey.

### 2.5. Dough Measurements

#### 2.5.1. Rheofermentometer Analysis

A rheofermentometer F3 (Chopin, Villeneuve-La-Garenne, France) was used to assess the fermentation characteristics of the doughs containing the different percentages of honey. Both dough development and carbon dioxide production and retention curves were used. An amount of 315 g of dough was prepared using a planetary mixer (KitchenAid Professional 4.8 L, model 5KSM175PS, St. Joseph, MI, USA), equipped with a flexible edge flat beater (5KFE5T), then incubated for 180 min at a temperature of 28.5 °C in a fermentation chamber and covered with a stainless-steel cylinder without any additional weight. The rheofermentometer curves were used to give: the maximum dough development height (Hm, in mm); the time taken to achieve maximum dough rise (T_1_, in min); the final dough height at the end of the test (h, in mm); the percentage decrease in dough volume at the end of the test ((Hm-h)/Hm, in %); the maximum height of gaseous release (H’m, in mm); the total amount of gas produced (V_TOT_, in mL); the amount of gas retained (V_RET_, in mL); the amount of gas released (V_REL_, in mL); and the gas retention coefficient (RC), calculated as V_RET_/V_TOT_ (%). To assess the amylase activity, the stirring number was determined following the methodology reported in the manufacturer manual. The tests were performed twice for each dough formulation.

#### 2.5.2. Doughs Viscometric Properties

The viscometric properties of the GF doughs produced were assessed using a Rapid Visco Analyser (RVA-4, Newport Scientific, Warriewood, Australia) in accordance with the method suggested by the manufacturer. Pasting profiles were obtained. The pasting temperature (°C), peak time (when peak viscosity occurred; min), peak viscosity (maximum hot paste viscosity), breakdown (peak viscosity minus holding strength or minimum hot paste viscosity), setback (final viscosity minus holding strength), and final viscosity (end of the test after cooling to 50 °C and holding at this temperature) were calculated. All the viscosity parameters are expressed in mPa. Dough samples were prepared by dispersing 3.5 g of each formulation, as reported in Section 2.2, in 25 mL of distilled water into an aluminum canister. The doughs were then subjected to heating and cooling following these steps: holding at 50 °C for 1 min, gradual heating to 95 °C, holding at 95 °C for 2 min 30 s, cooling to 50 °C, and lastly holding at 50 °C for 2 min. The test was performed twice for each dough formulation.

#### 2.5.3. Small Deformation Characteristics

To determine the rheological properties of the dough, an oscillation test was performed using a dynamic shear rheometer (Anton Paar MCR 92, GmbH Inc., Graz, Austria) equipped with a 50 mm plate (P50/P2). The dough samples were prepared without yeast and allowed to rest at room temperature for 20 min before analysis. Around 2 g of dough core was taken using a spatula and placed between the plates for 2 min to relax, with the upper plate lowered against the sample to achieve a dough thickness of 2 mm. A thin layer of paraffin oil was applied to the sample edge to prevent moisture loss. The determination of the linear viscoelastic region (LVR) of the dough samples was carried out at a constant frequency value of 10 Hz and at strain values that varied from 0.001 to 100 s^−1^. From the profiles obtained, it was possible to determine the critical value of the shear stress corresponding to the point at which the elastic modulus G’ began to deviate from the previously constant values. Therefore, based on the results obtained, it was decided to use a strain value of 0.01% (within the LVR) to determine the viscoelastic properties of the mixtures. The dynamic frequency sweep test was then conducted by keeping the shear stress value constant and varying the deformation frequency (0.1 to 10 Hz). Each dough formulation was prepared twice, and each sample measured at least three times.

### 2.6. Bread Measurements

#### 2.6.1. Weight Determination

Baked samples were left to cool and subsequently weighed using a technical scale.

#### 2.6.2. Determination of Specific Volume

Volume determination was performed using the official rapeseed displacement AACC method 10-05.01 [31]. The specific volume was calculated as the ratio between the volume expressed in mL and the weight expressed in g of each loaf. Each batch was subjected to triplicate determinations for each sample.

#### 2.6.3. Determination of Moisture Content

The moisture content of the breads was calculated in accordance with the official standard method AACC 44-15A [31] with some modifications. This method, which comprises two consecutive stages, entails determining the quantity of water as weight loss of a sample heated under specific conditions. In the initial phase, a representative portion of three breads per batch was cut into uniform cubes and left to dry for 48 h in a ventilated and heated environment. The resulting weight is then expressed as a percentage value and indicated as the humidity of the first stage. The partially dried samples obtained from the first stage are subsequently ground for the determination of the humidity of the second stage. For this purpose, approximately 3 g of sample is weighed and subsequently analyzed using a thermobalance (KERN & SOHN GmbH, Model Kern-DAB 100-3, Balingen, Germany). 

The total moisture content of the samples was calculated using the following formula:

Htot (%) = A + (B × (100 − A))/100(1)

where A corresponds to the moisture value obtained in the first stage and B corresponds to the moisture value measured in the second stage. Each batch was subjected to triplicate determinations for each sample. 

#### 2.6.4. Color of the Crust and Crumb

The color of crust and crumb was determined using a tristimulus colorimeter coupled to a CR-300 measuring unit (Minolta, Japan), which was previously calibrated against a white tile. The results were expressed according to the Hunter Lab color space, and the parameters acquired were lightness L*, redness (a*), and yellowness (b*). The color of the crust was determined at three different points on the bread surface (middle and end), with three different loaves evaluated for each sample per batch. The color of the crumb was assessed on a central slice of each bread sample. In addition to the colorimetric parameters, a secondary index was calculated, the crust browning index (BI), obtained by subtracting the brightness value L from 100.

#### 2.6.5. Texture Profile Analysis (TPA)

To evaluate the rheological properties of the finished product, the bread samples were subjected to texture profile analysis (TPA), which was conducted using a texturimeter (TA-XT2 Texture Analyzer, Stable Microsystem, Surrey, UK). The sample was subjected to a double compression cycle, which simulates the chewing process in the mouth. This analysis allows for the identification of the primary mechanical properties (hardness, elasticity, cohesiveness, and resilience) and the secondary attribute (chewiness). 

The analysis was performed on the central portion of 2 slices of bread with a thickness of 2 cm, taken from the central region of three different loaves of bread for each sample. The bread slices were compressed and released twice with the assistance of a cylindrical aluminum probe with a diameter of 25 mm and a time of 30 s between each compression. The samples were compressed to 50% of their initial thickness with a probe test speed of 1 mm/s. The resistance was recorded on a force–time diagram (N/s), from which the following primary and secondary mechanical properties of the bread were obtained: hardness, as the maximum peak force (N) to break the sample; cohesiveness, as the ratio between the area delimited by the curve representing the compression of the second cycle, divided by that of the first cycle; springiness (elasticity), which represents the time that the food takes to recover its initial height between one compression and another; resilience (recovery capacity), as the ratio between the area under the curve at the moment in which the compression force ceases and the area under the curve during the first compression; and chewiness, a secondary parameter obtained from the product of hardness, cohesiveness, and springiness.

### 2.7. Statistical Analysis

The statistical analysis of the data was carried out using the Statistica 10 software (StatSoft, Inc., Tulsa, OK, USA). The data obtained experimentally were analyzed using one-way analysis of variance (ANOVA), in which the categorical predictor was represented by the type of honey substituted for sucrose in the preparation of the samples. To evaluate the difference between the sample means, Fisher’s LSD test was applied using a 95% confidence interval (*p* < 0.05).

## 3. Results and Discussion

### 3.1. Moisture Content of Honeys

Before discussing the results of physical–chemical and technological properties of the doughs and breads, it seems necessary to report the water content of the different types of honey used during the experiment. Table 1 reports Brix degrees, refractive index, and moisture content of the samples used. 

As reported in Annex II of the Directive 2001/110/EC of the Council of the European Union, honey, “when placed on the market as such and used in products intended for ab consumption”, must have a moisture content not exceeding 20% [32]. 

As can be seen from the data reported in Table 1, all the types of honey studied have moisture values falling within the limits established by law.

In particular, with the exception of EU, in which the lowest humidity value was observed (15%), all the samples showed a water content between 17.13% of ST and 18.10% of MF (used as a reference value).

It should be emphasized that, in order to maintain a constant sugar concentration in all the experimental breads, the exact percentage of sucrose replacement was calculated with each of the honeys used, taking into account the humidity value present in each of them and using the water content of the MF as a reference (Table 2).

### 3.2. Glucose and Fructose Content of the Honey Samples

The glucose and fructose content, as well as the fructose/glucose ratio, of the honey samples are reported in Table 3. Honey is composed primarily of sugars, which constitute approximately 70–80% of the total composition and over 95% of the dry substance. Approximately 90% of the total sugars present in honey are glucose and fructose, which are frequently present in similar percentages of 40% and 30%, respectively [33]. A further 5–10% is made up of other sugars (more than 20 have been identified), but, among these, only sucrose is present in significant quantities in compositional terms [34]. Fructose has been reported to be the main sugar in honey, with a few exceptions, such as in rape (*Brassica napus*), dandelion (*Taraxacum officinale*), and blue curl (*Trihostema lanceolatum*) [35]. The present data are in agreement with this, as the fructose levels were always higher than the glucose levels in all five different honeys. The MF sample has the highest fructose content and the lowest glucose content compared to the other samples. The fructose/glucose ratio varied from 1.11 for ST to 1.53 for MF. This value is important because a fructose to glucose ratio equal to or lower than 1.14 may result in fast granulation, while there is no tendency to granulation when values are higher than 1.58 [36,37].

### 3.3. Dough Characterization

#### 3.3.1. Leavening Properties and Amylase Activity

The data obtained through rheofermentometric analysis are reported in Table 4. All gluten-free doughs prepared by replacing sucrose with different types of honey showed good development curves, which did not differ significantly from the behavior of the CTRL containing sucrose alone. Indeed, the supplemented samples, although they showed, in some cases, a less rapid development, did not exhibit statistically significant differences (*p* < 0.05) compared to the CTRL in terms of both Hm and h (Table 4). However, the analysis of the data related to the decrease in the final volume of the dough (Hm-h)/Hm revealed that some supplemented doughs exhibited greater and more prolonged stability compared to the CTRL. In particular, the partial replacement of sucrose with EU and HE, as well as the total replacement of sucrose with ST and EU, resulted in a significantly lower reduction in the final volume of the dough with respect to the CTRL sample (Table 4). The greater stability shown by the aforementioned samples could be due to a better combination between the gases produced and the microstructural properties of the system, which would contribute to maintaining the macrostructure of the dough during leavening [38]. Among other supplemented doughs, the highest final volume reduction values were recorded in those prepared by using the MF at 5% level.

In contrast with the observations made regarding the development curve, the behavior of the majority of the supplemented doughs was found to be significantly different from that of the CTRL in terms of the quantity of carbon dioxide produced and released during the fermentation process. In particular, all the supplemented samples, with the exception of those prepared with partial substitution of sucrose with CA and EU and those prepared with partial or total replacement of sucrose with MF, showed a significant reduction in the total amount of CO_2_ produced (but also a lower amount of CO_2_ released), compared to the CTRL sample. The reasons for this different behavior are probably linked to a different sugar composition of honey, which, in some cases, may have been more favorable and readily available for yeast metabolism. The lowest levels of CO_2_ produced were observed in the samples prepared with the high levels of sucrose replacement with ST (1094 mL) and HE (1124 mL). It is known that, during the leavening process, in addition to the total quantity of CO_2_ produced, the ability of the dough to retain the gas produced is a crucial factor influencing not only the quantity of gases produced and released by the system but also the quality of the finished product. In the experimental doughs, with the exception of EU2.5%, the samples with a greater total CO_2_ production recorded the lowest retention coefficients, indicating a probable reduction in viscoelastic capacity compared to the other samples. Conversely, the doughs with a lower production of total CO_2_ exhibited high values of the retention coefficient, probably suggesting superior microstructural properties and enhanced structural stability. The substitution of sucrose resulted in a reduction in the stirring number, with some exceptions (MF2.5% and EU5%) in comparison to the CTRL. A comprehensive examination of the data obtained during the reofermentographic test indicates that the most optimal fermentation performances were achieved through the partial substitution of sucrose with EU, as well as through the partial or total replacement with MF.

To the best of the authors knowledge, this is the first study to investigate the impact of substituting sugar with honey on leavening properties and amylase activity of doughs.

#### 3.3.2. Viscometric Properties of the Doughs

The RVA data of the flour mixtures are reported in Table 5. 

The partial or total substitution of sugar with honey led to a significant decrease in peak viscosity, breakdown, and final viscosity, which is consistent with the findings of [39]. However, it should be noted that their study focused on gluten-containing bread. The greater hygroscopic nature of honey with respect to sucrose may have competed with water that was less available for gelatinization, thus resulting in a lower peak viscosity. This is further demonstrated by the fact that an increase in the rate of sugar replacement resulted in a slight decline in the peak viscosity values, though the change was not statistically significant. A reduction in water availability may also explain the observed decrease in breakdown, as it depends on the minor water available, which in turn reduced the starch breakdown. 

The setback value, which results from an increase in viscosity during the cooling period, indicates the tendency of the amylopectin to re-associate with a decrease in temperature. It is inversely associated with the retrogradation phenomenon. In the present study, the substitution of sugar with EU and HE and the total substitution with CA and ST resulted in lower values than the CTRL, indicating a reduced possibility for starch retrogradation.

#### 3.3.3. Small Deformation Characteristics

The values of the storage modulus (G′) were higher than the values of the loss modulus (G″) in all the samples analyzed, suggesting a solid, elastic-like behavior for all the experimental mixtures, including the CTRL (Table 6).

The partial replacement of sucrose with CA, MF, and ST, as well as the total replacement of sucrose with CA, had no influence on the viscoelastic properties of the supplemented doughs (Table 6). In contrast, the other samples (EU2.5% and 5%, HE2.5% and 5%, and MF5% and ST5%) resulted in higher G′ and G″ values with respect to CTRL. It is noteworthy that these findings aligned with those obtained through reofermentographic analysis, with the exception of the HE2.5%, HE5%, and ST5% samples. The ability of a dough (and, therefore, of its structure) to produce, retain, and stabilize the CO_2_ produced during fermentation is contingent upon three primary factors: (a) the carbohydrate composition, (b), the viscoelastic properties of the system, and (c) the combination of these two factors (whether more or less favorable). In the ST5% and EU5% doughs, for example, the lower volume of CO_2_ produced during leavening was compensated by the better viscoelastic characteristics of the system, which allowed maximum development of the dough, very similar to that of the CTRL, but with significantly higher values of both stability and ability to retain the gases produced (however significantly lower) during leavening (Table 4 and Table 6).The reduction in the percentage of EU added to the basic formulation did not influence the viscoelastic properties of the system, suggesting that, in this case, the better leavening performances observed were the result of an optimal combination between the sugar composition and the microstructural properties of the experimental dough. Contrasting results were obtained for the HE2.5%, HE5%, and MF5% samples, which, despite exhibiting superior viscoelastic properties, showed a CO_2_ retention capacity similar to that observed in the CTRL sample (Table 6). Furthermore, it is to highlight that an increase in sucrose substitution from 50% to 100% led to an improvement in the viscoelastic properties of all the doughs with the exception of HE and EU.

To the best of the authors knowledge, these are the first reported results on the influence of sugar substitution with honey on the small deformation characteristics of doughs.

### 3.4. Bread Quality

#### 3.4.1. Specific Volume

**Figure 1:**
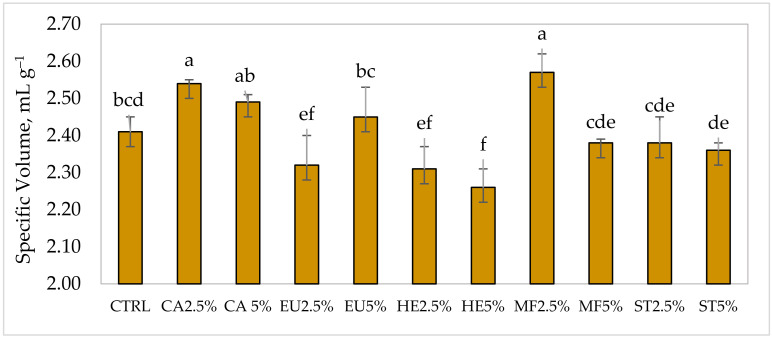
Specific volume of control and honey-enriched bread samples (*n* = three per batch). Bars of histograms with the same letters do not differ significantly from each other according to LSD test (*p* < 0.05). CTRL: control; CA: cardoon; EU: eucalyptus; HE: honeydew eucalyptus; MF: multifloral; ST: strawberry tree.

One of the main visual characteristics of bread that influences consumers, and their purchasing decisions, is represented by the specific volume. High values of this parameter are often associated with adequate gas production during fermentation, which translates into an aerated and open structure of the finished product. The bread samples under study showed specific volume values that varied from 2.26 ± 0.05 mL g^−1^ of the sample prepared by completely replacing the sucrose with HE5% to 2.57 ± 0.06 mL g^−1^ of the sample prepared by completely replacing the sucrose with MF2.5% (Figure 1). 

An intermediate specific volume, equal to 2.42 ± 0.04 mL g^−1^, was instead observed in the CTRL sample, which contained sucrose alone. The observed values fall within the range of specific volume typically reported in the literature for gluten-free bread of the loaf type [29,40].

In particular, among the supplemented breads, the highest specific volume values, which were significantly different from CTRL, were observed for the MF2.5% and CA2.5% breads. The partial replacement of sucrose with EU as well as the partial or total replacement of the same with HE resulted in a significant reduction in the specific volume of supplemented breads in comparison to the CTRL sample. 

The literature reports that Hm is a reliable indicator of the specific volume values observed in the corresponding finished products. In fact, as an indirect estimate of the fermentative activity of the yeasts and of the microstructure created in the system during leavening, a high Hm value should lead to high specific volume values [41,42]. 

However, in the present study, data indicated that there were no significant differences in HM values among the samples. Therefore, the extreme variability in the specific volume values observed between the supplemented samples may depend on other factors. In particular, all the breads with specific volume values significantly higher than the CTRL were obtained from doughs with the highest retention volumes, indicating better ventilation of the system. The only exception is the EU2.5% sample, which exhibited good development curves, excellent stability, and a high capacity for retaining the gases produced. However, it resulted in a bread with a significantly lower specific volume value than the CTRL sample (Figure 1).

#### 3.4.2. Crust and Crumb Color

The partial or total replacement of sucrose with the different types of honey had a significant influence on the color of the crust of all the supplemented breads (Table 7). 

The statistical analysis of the data revealed that all the samples supplemented with honey showed higher brightness values (L) compared to the control, with the exception of the MF5% sample. Additionally, the samples exhibited lower values of the red component of the color (positive values of the a* coordinate), with the exception of EU2.5% and MF5%, and lower values of the index of browning compared to the CTRL sample, with the exception of MF5%, suggesting a lighter color of the honey-supplemented samples. 

These results are probably due to a different composition of the sugar substance present in the different types of honey. In fact, it is known that the browning observed in baked products during the cooking phase is the result of a complex series of reactions, including the Maillard reaction between reducing sugars and amino acids present and the caramelization reaction of the sugars. These reactions lead to the formation of highly pigmented, high molecular weight compounds, known as melanoidins, which are responsible for the appearance of the red/brown color of the finished product. Furthermore, considering that the nature of the reducing sugars present in food matrices represents a primary factor influencing both the appearance and the speed of these reactions (with fructose, for instance, being more reactive than glucose), it would be reasonable to expect a greater browning effect in samples with the total or partial replacement of honey. These results are in contrast with those reported in other studies, wherein the replacement of sucrose with honey powder [28] or liquid honey [27] was observed to positively impact all colorimetric indices of the crust of soft wheat breads. The authors in question attributed this effect to the greater quantity of fructose and glucose introduced with honey in the formulation of the breads, which, as is known, represent one of the main factors capable of influencing the appearance and intensity of non-enzymatic browning reactions. However, the present results are in accordance with those reported by [39], who found a significant lightening of the crust in bread samples when sucrose was entirely replaced with liquid honey, while a browning effect was seen in bread samples where sucrose was substituted with powdered honey. Thus, it seems that the state of the honey used (MF powder vs. fresh honey) drives the changes in color. The fact that the use of liquid honey results in a lightening of the crust may be tentatively explained by considering the higher water content of honey with respect to sucrose, which resulted in breads with higher moisture content compared to the CTRL (Table 8) and therefore a probable higher water activity in the corresponding samples, with a consequent shift towards a range where the non-enzymatic browning reaction is less favorable. Moreover, honey contains different polyphenols, such as phenolic acids and flavonoids [43], which can effectively reduce the non-enzymatic browning reactions [44]. 

The partial or total replacement of sucrose with the different types of honey influenced the color of the crumb of the experimental breads to a much lesser extent than what was observed for the color of the crust (Table 7). In particular, the parameter L was not influenced, except for CA5% and HE2.5%, the latter being also the only sample statistically different from CTRL for the a* parameter. The L values were very similar to those reported by [27]. On the other hand, more differences were found for the coordinate b*, which was statistically higher, indicating a more yellow color of the crumb, in samples prepared with CA and HE and in ST2.5%. The results obtained for b* are in agreement with those reported by [28] and by [39] for samples with liquid honey. In the work of [27], on the other hand, for the substitution of sugar, only liquid honey was used.

#### 3.4.3. Texture Profile

The replacement of sucrose with honey produced variable effects on the texture characteristics of the experimental breads, depending on both the type of honey used and the percentage of substitution applied (Table 8).

The samples prepared with both percentages of ST and sample HE2.5% exhibited an increase in hardness compared to the CTRL, while samples prepared with both percentages of CA and samples EU5% and MF2.5% showed a decrease. The positive softening effect on the bread could be attributed to the greater quantity of fructose (which is more hygroscopic than sucrose) present in these types of honey compared to both sucrose and the other types of honey used in the experiment. A similar positive softening effect was reported by [28] when replacing up to 15% of sucrose with powdered honey. 

Furthermore, the samples prepared with both percentages of CA and sample MF2.5% showed significantly lower chewiness values (a parameter linked to hardness by a direct proportionality) compared to the CTRL, as also reported by [28]. It is also important to underline that the types of honey and related substitution percentages, which had a positive effect on the softness of the bread, were the same ones that resulted in the highest specific volume (Figure 1).

With regard to springiness, which represents the recovery capacity of the sample when the applied force is removed, no statistical difference was observed between the samples with honey and the CTRL, with the exception of CA2.5%.

The effects linked to the replacement of sucrose with the various types of honey on cohesiveness values followed the same trend observed for the other texture parameters. In fact, the lowest cohesiveness values were recorded in samples with the highest hardness values (ST). Conversely, the highest cohesiveness values were obtained in the breads with greater softness and elasticity. In this case, the data differ from those reported by [28]. 

Finally, the samples with the highest hardness (ST2.5% and ST5%) exhibited significantly lower resilience compared to the CTRL. Conversely, the CA2.5% sample, which had the lowest hardness value, displayed the opposite trend.

## 4. Conclusions

The findings of the present study indicate that the impact of substituting sucrose with various types of honey on the technological quality of gluten-free bread is more closely associated with the specific type of honey utilized than with the degree of substitution employed. In particular, doughs prepared with partial sucrose replacement with CA and MF, as well as those prepared with total replacement with CA, exhibited comparable fermentation performances and viscoelastic characteristics to the CTRL sample. However, they yielded finished products with higher specific volume and greater softness of the crumb. It is noteworthy that the CA2.5% sample also showed greater elasticity and cohesiveness. In contrast, the replacement of sucrose with ST and HE resulted in the least favorable outcomes at both the dough and finished product levels. All other samples showed intermediate characteristics. One potential issue might be related to the browning of the crust, which was observed to be less intense in all supplemented samples compared to the CTRL. In conclusion, the results obtained revealed that the use of specific types of honey could be a promising strategy to produce gluten-free breads that, while maintaining their technological quality characteristics, meet healthy criteria by reducing the amount of added sugars by 50%. 

## Figures and Tables

**Table 1:** Refractive index and moisture content of the honey samples.

Samples ^1^	Brix Degrees (%)	Refractive Index (nD^2^⁰)	Moisture (g 100 g^−1^)
CA	81.17 ± 0.29 b	1.494 ± 0.000 b	17.20 ± 0.17 b
EU	83.37 ± 0.31 a	1.499 ± 0.001 a	15.00 ± 0.17 c
HE	81.07 ± 0.29 b	1.493 ± 0.001 b	17.27 ± 0.23 b
MF	80.23 ± 0.31 c	1.491 ± 0.001 c	18.10 ± 0.26 a
ST	81.33 ± 0.06 b	1.494 ± 0.000 b	17.13 ± 0.12 b

^1^ Mean values ± standard deviation. Within columns, values (mean of three replicates) with the same letter do not differ significantly from each other according to LSD test (*p* < 0.05). CA: cardoon; EU: eucalyptus; HE: honeydew eucalyptus; MF: multifloral; ST: strawberry tree.

**Table 2:** Sucrose replacement percentages of experimental bread formulations corrected for moisture content of each honey.

Samples	Honey (%)	Sucrose (%)
CTRL	0	5
CA2.5%	2.4	2.5
CA5%	4.8	0
EU2.5%	2.1	2.5
EU5%	4.1	0
HE2.5%	2.4	2.5
HE5%	4.8	0
MF2.5%	2.5	2.5
MF5%	5	0
ST2.5%	2.4	2.5
ST5%	4.7	0

CTRL: control; CA: cardoon; EU: eucalyptus; HE: honeydew eucalyptus; MF: multifloral; ST: strawberry tree.

**Table 3:** Glucose and fructose content of honey samples (g 100 g^−1^).

Samples ^1^	Glucose (g 100 g^−1^)	Fructose (g 100 g^−1^)	Fructose/Glucose Ratio
CA	31.59 ± 0.80 b	39.81 ± 0.44 b	1.26 ± 0.05 b
EU	29.96 ± 0.04 c	37.34 ± 0.07 cd	1.25 ± 0.00 b
HE	28.42 ± 0.14 d	36.33 ± 0.24 d	1.28 ± 0.00 b
MF	27.73 ± 0.52 d	42.51 ± 0.71 a	1.53 ± 0.00 a
ST	33.82 ± 0.17 a	37.71 ± 0.30 c	1.11 ± 0.00 c

^1^ Mean values ± standard deviation. Within columns, values (*n* = 3) with the same letter do not differ significantly from each other according to LSD test (*p* < 0.05). CA: cardoon; EU: eucalyptus; HE: honeydew eucalyptus; MF: multifloral; ST: strawberry tree.

**Table 4:** Leavening properties and amylase activity of control and honey-enriched dough samples.

Samples ^1^	Dough Development Parameters				Gas Release Parameters						
	H_m_ (mm)	T_1_ (min)	h (mm)	(H_m_-h)/H_m_ (%)	H′_m_ (mm)	T_X_ (min)	V_TOT_ (mL)	V_REL_ (mL)	V_RET_ (mL)	RC (%)	Stirring Number (RVU)
CTRL	73 ± 1 a	107.3 ± 3.2 cd	70.4 ± 1.3 a	3.5 ± 0 b	84 ± 0.1 abc	72.0 ± 4.2 a	1344 ± 13 a	51.5 ± 0.7 a	1293 ± 13 a	96.2 ± 0.1 e	169 ± 3 a
CA2.5%	69 ± 5.0 a	107.3 ± 3.2 cd	66.6 ± 4.9 a	2.9 ± 0 b	83.3 ± 3.3 abc	71.3 ± 1.1 a	1313 ± 42 abc	50.5 ± 4.9 a	1262 ± 37 ab	96.2 ± 0.3 e	147 ± 4 f
CA5%	63.1 ± 1.2 a	108.8 ± 3.2 c	61.1 ± 0.8 a	3.1 ± 0.5 b	81.9 ± 1.5 abc	74.3 ± 5.3 a	1255 ± 19 bcd	36.5 ± 3.5 b	1217 ± 16 ab	97.1 ± 0.3 d	151 ± 3 ef
EU2.5%	71.3 ± 3.8 a	111.0 ± 0.0 bc	69.6 ± 4.5 a	2.5 ± 1.1 b	82.9 ± 1.6 abc	75.8 ± 7.4 a	1298 ± 7 abcd	38.0 ± 5.7 b	1260 ± 13 ab	97.1 ± 0.5 d	161 ± 1 bcd
EU5%	70.6 ± 3.1 a	120.0 ± 0.0 a	70.6 ± 3.1 a	0 c	79.9 ± 4.7 cd	75.0 ± 8.5 a	1144 ± 82 ef	22.5 ± 3.5 cd	1121 ± 79 cd	98.1 ± 0.2 bc	170 ± 0 a
HE2.5%	72.2 ± 1.2 a	120.0 ± 0.0 a	72.2 ± 1.2 a	0 c	80 ± 1.4 bcd	80.3 ± 1.1 a	1230 ± 19 cd	25.0 ± 1.4 cd	1205 ± 18 b	98.0 ± 0.1 bc	158 ± 3 cd
HE5%	66.1 ± 0.7 a	120.0 ± 0.0 a	66.1 ± 0.7 a	0 c	77.2 ± 2.0 d	75.8 ± 1.1 a	1124 ± 65 f	19.0 ± 4.2 de	1106 ± 60 cd	98.4 ± 0.4 ab	163 ± 4 bc
MF2.5%	71.0 ± 4.3 a	108.0 ± 0.0 c	68.5 ± 4.5 a	3.5 ± 0.5 b	84.6 ± 1.9 ab	69.8 ± 1.1 a	1336 ± 20 ab	49.0 ± 0.0 a	1287 ± 20 a	96.4 ± 0.1 e	165 ± 2 ab
MF5%	70.01 ± 1.2 a	101.3 ± 5.3 d	66.4 ± 1.3 a	5.2 ± 0.3 a	86.2 ± 0.8 a	68.3 ± 7.4 a	1303 ± 6 abc	48.0 ± 2.8 a	1255 ± 3 ab	96.3 ± 0.2 e	159 ± 3 cd
ST2.5%	67.6 ± 6.2 a	116.3 ± 5.3 ab	67.1 ± 6.9 a	0.8 ± 1.1 c	79.7 ± 0.0 cd	79.5 ± 4.2 a	1213 ± 40 de	27.0 ± 0.0 c	1186 ± 40 bc	97.8 ± 0.1 c	153 ± 0 e
ST5%	66.3 ± 0.3 a	120.0 ± 0.0 a	66.3 ± 0.3 a	0 c	76.1 ± 1.2 d	85.5 ± 2.1 a	1094 ± 28 f	13.5 ± 2.1 e	1081 ± 26 d	98.8 ± 0.2 a	156 ± 0 de

^1^ Mean values ± standard deviation. Within columns, values (*n* = two per batch) with the same letter do not differ significantly from each other according to LSD test (*p* < 0.05). CTRL: control; CA: cardoon; EU: eucalyptus; HE: honeydew eucalyptus; MF: multifloral; ST: strawberry tree.

**Table 5:** Pasting properties of control and honey-enriched dough samples.

Samples ^1^	Pasting Temperature (°C)	Peak Time (min)	Peak Viscosity (Pa × s)	Breakdown (Pa × s)	Setback (Pa × s)	Final Viscosity (Pa × s)
CTRL	79 ± 2 a	6.1 ± 0.1 a	2354 ± 23 a	467 ± 16 a	1147 ± 41 a	3034 ± 48 a
CA2.5%	80 ± 1 a	6.1 ± 0 a	2054 ± 51 de	362 ± 47 b	1033 ± 25 abcd	2726 ± 124 d
CA5%	81 ± 1 a	6.3 ± 0.1 a	1976 ± 34 e	332 ± 59 b	952 ± 61 d	2597 ± 36 e
EU2.5%	81 ± 0 a	6.2 ± 0.1 a	2082 ± 67 cd	355 ± 65 b	993 ± 68 bcd	2720 ± 70 d
EU5%	81 ± 2 a	6.2 ± 0.1 a	2067 ± 33 d	335 ± 1 b	946 ± 45 d	2679 ± 13 de
HE2.5%	81 ± 1 a	6.2 ± 0.2 a	2168 ± 4 bc	390 ± 5 ab	993 ± 16 bcd	2771 ± 17 cd
HE5%	80 ± 2 a	6.2 ± 0.1 a	2089 ± 18 cd	332 ± 64 b	943 ± 71 d	2700 ± 25 de
MF2.5%	80 ± 2 a	6.2 ± 0.1 a	2071 ± 40 d	316 ± 39 bc	1099 ± 51 ab	2854 ± 52 bc
MF5%	80 ± 2 a	6.2 ± 0.1 a	2022 ± 38 de	226 ± 56 c	1071 ± 16 abc	2867 ± 1 bc
ST2.5%	79 ± 0 a	6.2 ± 0.1 a	2198 ± 66 b	361 ± 21 b	1079 ± 0 abc	2916 ± 45 b
ST5%	77 ± 5 a	6.3 ± 0 a	2024 ± 9 de	330 ± 67 b	977 ± 93 cd	2671 ± 35 de

^1^ Mean values ± standard deviation. Within columns, values (*n* = two per batch) with the same letter do not differ significantly from each other according to LSD test (*p* < 0.05). CTRL: control; CA: cardoon; EU: eucalyptus; HE: honeydew eucalyptus; MF: multifloral; ST: strawberry tree.

**Table 6:** Viscoelastic properties (Pa) of control and honey-enriched dough samples.

Samples ^1^	Storage Modulus G′	Loss Modulus G″	tan *δ*
CTRL	4076 ± 354 d	1379 ± 100 e	0.339 ± 0.005 a
CA2.5%	4774 ± 287 cd	1546 ± 2 cde	0.324 ± 0.02 a
CA5%	4834 ± 583 cd	1562 ± 150 cde	0.324 ± 0.008 a
EU2.5%	5701 ± 147 ab	1826 ± 129 abc	0.320 ± 0.014 a
EU5%	5404 ± 435 bc	1683 ± 84 bcd	0.312 ± 0.010 a
HE2.5%	6217 ± 359 a	1876 ± 305 ab	0.301 ± 0.032 a
HE5%	5927 ± 575 ab	1991 ± 206 a	0.336 ± 0.002 a
MF2.5%	4778 ± 118 cd	1550 ± 27 cde	0.325 ± 0.002 a
MF5%	5834 ± 118 ab	1823 ± 19 abc	0.313 ± 0.009 a
ST2.5%	4182 ± 19 d	1448 ± 21 de	0.347 ± 0.004 a
ST5%	5718 ± 464 ab	1746 ± 22 abc	0.306 ± 0.021 a

^1^ Mean values ± standard deviation. Within columns, values (*n* = three per batch) with the same letter do not differ significantly from each other according to LSD test (*p* < 0.05). CTRL: control; CA: cardoon; EU: eucalyptus; HE: honeydew eucalyptus; MF: multifloral; ST: strawberry tree.

**Table 7:** Crust and crumb color parameter of control and honey-enriched bread sample.

Samples ^1^	Crust Color				Crumb Color		
	L_cs_*	a_cs_*	b_cs_*	BI ^2^	L_cb_	a_cb_*	b_cb_*
CTRL	54 ± 1 e	12.6 ± 0.4 ab	32 ± 0 cd	46 ± 1 a	64 ± 0 bcd	0.4 ± 0 bcd	5.4 ± 0.5 d
CA2.5%	60 ± 1 c	9.7 ± 0.4 de	30 ± 1 de	40 ± 1 c	64 ± 1 bcde	0.5 ± 0 abc	6.1 ± 0.6 abc
CA5%	68 ± 0 b	5.2 ± 0.9 fg	23 ± 2 h	32 ± 0 d	67 ± 0 a	0.7 ± 0.2 ab	6.3 ± 0.1 ab
EU2.5%	58 ± 0 d	11.7 ± 0.3 bc	34 ± 0 bc	42 ± 0 b	65 ± 0 abc	0.2 ± 0.1 d	5.6 ± 0 cd
EU5%	69 ± 2 ab	4.7 ± 0.1 g	24 ± 1 gh	31 ± 2 de	64 ± 2 bcd	0.3 ± 0.1 cd	5.8 ± 0.3 bcd
HE2.5%	69 ± 1 ab	6.1 ± 0.2 f	27 ± 1 f	31 ± 1 de	62 ± 1 e	0.7 ± 0.1 a	6.2 ± 0 abc
HE5%	58 ± 0 d	11.5 ± 0.1 c	35 ± 0 a	42 ± 0 b	63 ± 1 cde	0.2 ± 0 d	6.2 ± 0.1 abc
MF2.5%	57 ± 1 d	10.2 ± 0.7 d	30 ± 0 e	43 ± 1 b	63 ± 0 de	0.2 ± 0.1 d	5.3 ± 0.1 d
MF5%	55 ± 2 e	13 ± 1.2 a	35 ± 0 ab	45 ± 2 a	63 ± 1 de	0.1 ± 0.1 d	5.8 ± 0.1 bcd
ST2.5%	71 ± 1 a	4.9 ± 0.3 g	25 ± 1 fg	29 ± 1 e	63 ± 1 de	0.4 ± 0.4 abcd	5.2 ± 0.4 d
ST5%	61 ± 1 c	9.1 ± 0.6 e	31 ± 1 de	39 ± 1 c	65 ± 0 ab	0.3 ± 0.1 cd	6.4 ± 0.1 a

^1^ Mean values ± standard deviation. Within columns, values (*n* = three per batch) with the same letter do not differ significantly from each other according to LSD test (*p* < 0.05). CTRL: control; CA: cardoon; EU: eucalyptus; HE: honeydew eucalyptus; MF: multifloral; ST: strawberry tree; ^2^ BI: browning index.

**Table 8:** Textural properties and moisture content of control and honey-enriched bread samples.

Samples ^1^	Textural Properties					Moisture (g 100 g^−1^)
	Hardness (N)	Springiness	Cohesiveness	Chewiness	Resilience	
CTRL	4.17 ± 0.27 cd	0.99 ± 0 bc	0.69 ± 0.05 bcd	2.84 ± 0.15 bc	0.43 ± 0.04 bcd	35.4 ± 1.5 c
CA2.5%	3.26 ± 0.30 f	1.00 ± 0.01 a	0.75 ± 0.05 a	2.44 ± 0.05 e	0.47 ± 0.05 a	38.6 ± 0.9 b
CA5%	3.63 ± 0.18 ef	0.99 ± 0.01 abc	0.67 ± 0.02 cd	2.42 ± 0.17 e	0.41 ± 0.01 cde	39.3 ± 2 b
EU2.5%	4.45 ± 0.34 bc	0.99 ± 0.01 bc	0.66 ± 0.01 d	2.88 ± 0.23 bc	0.39 ± 0.01 def	38.7 ± 1.2 b
EU5%	3.54 ± 0.15 ef	1.00 ± 0.01 ab	0.73 ± 0.04 ab	2.57 ± 0.04 cde	0.46 ± 0.03 ab	41.3 ± 1.2 a
HE2.5%	4.9 ± 0.59 ab	0.98 ± 0.00 c	0.68 ± 0.00 cd	3.27 ± 0.40 a	0.4 ± 0.01 cde	39.2 ± 0.3 b
HE5%	3.91 ± 0.07 de	0.98 ± 0.00 c	0.65 ± 0.01 de	2.51 ± 0.09 de	0.38 ± 0.02 ef	40.1 ± 0.3 ab
MF2.5%	3.59 ± 0.1 ef	0.99 ± 0.01 abc	0.71 ± 0.03 abc	2.52 ± 0.13 de	0.44 ± 0.03 abc	39.7 ± 0.5 ab
MF5%	4.28 ± 0.24 cd	0.98 ± 0.01 c	0.66 ± 0.01 d	2.78 ± 0.21 bcd	0.39 ± 0.01 def	39.4 ± 1.1 b
ST2.5%	4.99 ± 0.31 a	0.98 ± 0.00 c	0.54 ± 0.00 f	2.66 ± 0.17 cde	0.29 ± 0.02 g	39.9 ± 0.5 ab
ST5%	4.95 ± 0.23 a	0.99 ± 0 abc	0.61 ± 0.02 de	2.97 ± 0.07 ab	0.35 ± 0.01 f	39.9 ± 1 ab

^1^ Mean values ± standard deviation. Within columns, values (*n* = six per batch) with the same letter do not differ significantly from each other according to LSD test (*p* < 0.05). CTRL: control; CA: cardoon; EU: eucalyptus; HE: honeydew eucalyptus; MF: multifloral; ST: strawberry tree.

## Data Availability

The original contributions presented in the study are included in the article, further inquiries can be directed to the corresponding author.
